# Evaluation of an undergraduate psychiatric clinical rotation: Exploring student perceptions

**DOI:** 10.4102/sajpsychiatry.v27i0.1583

**Published:** 2021-05-31

**Authors:** Inge M. Smit, Mariette Volschenk, Liezl Koen

**Affiliations:** 1 Department of Psychiatry, Faculty of Medicine and Health Sciences, Stellenbosch University, Cape Town, South Africa; 2 Centre for Health Professions Education, Faculty of Medicine and Health Sciences, Stellenbosch University, Cape Town, South Africa

**Keywords:** medical training, teaching and learning practices, medical students, undergraduate, clinical teachers, millennials

## Abstract

**Background:**

Globally, the appropriate transformation of medical training is critical to ensure the graduation of competent physicians who can address the growing health needs.

**Aim:**

To explore medical students’ perceptions of their learning experience during the undergraduate psychiatry late clinical rotation (PLCR) at Stellenbosch University (SU) and to use the findings to make possible recommendations regarding curriculum renewal.

**Setting:**

In recognition of this, the Department of Psychiatry at the Faculty of Medicine and Health Sciences of SU is reviewing its current teaching and learning practices.

**Methods:**

Data were collected from two focus groups.

**Results:**

Three main themes emerged: ‘learning in the clinical context’, ‘gaining knowledge’ and ‘generational needs’. Whilst several suggestions were made for potential improvement, the participants still endorsed that the PLCR does provide them with a good learning experience in psychiatry.

**Conclusions:**

Considering that these perceptions are from a group of millennials, the insights arising from the ‘generational needs’ theme were especially valuable. To bridge the generational gap and develop a curriculum that will not only meet the standards expected by educators but also achieve acceptance from learners, future research with a specific focus on clinical teachers’ perceptions is needed.

## Introduction

Globally, there has been an increasing focus on equipping medical graduates with the appropriate competencies to adequately address the growing health needs of society. This can be achieved by appropriately transforming medical training programmes.^1^ However, whilst designers of medical curricula should be fully cognisant of the emerging healthcare issues, they must simultaneously be mindful of the needs of the students.

**FIGURE 1:**
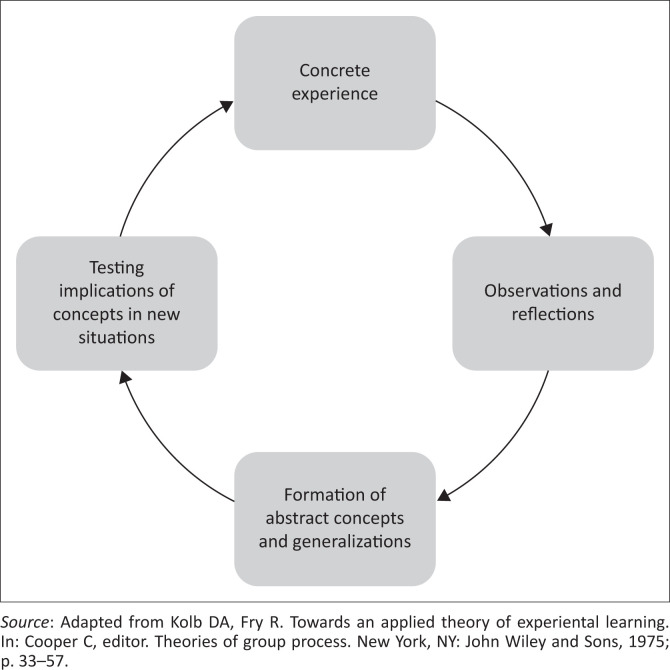
Kolb’s experiential learning model.

Harden^2^ describes the purpose of a ‘curriculum’ to be that of bringing order, coherence and intellectual discipline to the transmission of human experience. It is not simply the content that is taught, rather a curriculum should be designed to be specific and fit for purpose and be informed by the local political, cultural, professional and social contexts.^3^ Furthermore, a strong foundation in learning theories is needed to effectively design a curriculum.^4^ One such theory is that of experiential learning. Kolb^5^, described this as ‘the process whereby knowledge is created through the transformation of experience’, as illustrated in Figure 1.^6^ Knowledge and meaning are constructed from real-life experiences and interpersonal interactions in a context that is relevant to a student’s future career. Through participation in the workplace as well as reflection on this, the knowledge and meaning gained affect how new experiences are approached.^7^ Evaluation of curricula is then necessary to assess the extent to which a curriculum meets its purpose.^8^ By assessing the programme content, we are not only considering whether a curriculum ‘works’ but also gathering in-depth information on all aspects, including the possible unintended effects of the curriculum.^9^ The information gathered can then be used in a decision-making process as well as to give feedback to stakeholders.^8^

In reviewing a curriculum, the evaluators must be mindful of the interests of the students.^8^ The term ‘millennials’ was coined to refer to individuals born between 1982 and 2000.^10^ It could thus be inferred that the majority of current undergraduate medical students, as of the early 21st century, are of an age to be considered part of this generation. Millennials were born into a highly interconnected world and are accustomed to information being readily available, especially via technology. They are regarded as ‘confident’ and ‘motivated by self-interest’, possibly because of their upbringing by the so-called ‘helicopter parents’^10^; that is, parents who are overprotective or overinvolved in the lives of their children. They have a need for feedback, interaction with peers and a sense of accomplishment in their work.^11^ They value mentoring, personalised learning, working in teams and incorporating technology into learning.^10^ With regard to preferences and expectations, they generally have a different educational outlook in comparison to their earlier generation supervisors who often find it difficult to relate to them.

The Department of Psychiatry (DOP) at the Faculty of Medicine and Health Sciences (FMHS) of Stellenbosch University (SU) is reviewing its current teaching and learning practices. Whilst some data exist on using the perceptions of students regarding clinical rotations to improve curricula^12,13^; only one study has specifically investigated this in a general psychiatric undergraduate rotation in a South African setting. Their study used a self-developed questionnaire to gather information around students’ evaluation of their rotation and found that the overwhelming positive focus for the students was that of a friendly and relaxed learning environment.^14^ Our study used a qualitative method to determine the perceptions of students attending a current psychiatry rotation, the so-called psychiatry late clinical rotation (PLCR), at SU to formulate recommendations with a view to enhance the relevant curriculum components to graduate primary care physicians who are appropriately trained for mental health service delivery.^15^

## Methods

### Study design

This study used an exploratory, descriptive design generating data via thematic analysis. This type of research creates opportunities to generate theories of the world to be researched in an inductive manner.^16^

In this study, the ‘voice’ of the students as stakeholders^17^ was used to explore and understand the students’ perceptions of the PLCR at SU.

This was achieved by describing themes identified from these students’ dialogue.^16^

### Target population and sampling

The target population for this study was the 2018 final year MBChB student cohort at SU FMHS (*N* = 210). Students were divided into six groups and attended the PLCR sequentially throughout the year with larger groups in the latter third of the year. Our sample consisted of students from two sequential groups attending the PLCR in July and September 2018. In total, 97 students were there in these two groups. All were informed of the study and invited to partake through an electronic invite. Two focus groups were formed by using convenience sampling to include the first respondents who volunteered to participate.

### Data collection

Data were using two focus groups. This method has previously been shown to be valuable to evaluate medical education programmes.^18^

The groups consisted of seven (two male five female) and eight (two male and six female) participants. One interview per group was conducted by the primary researcher in a private location on campus, with each interview lasting approximately 50 min. Interviews were semi-structured and questions asked were guided by a document review of the existing psychiatry departmental data. After obtaining written and informed consent, both sessions were audio recorded and then transcribed independently. As the second focus group interview did not yield any new insights or thoughts, a third one was not indicated. During the interviews, the participants’ comments were reflected back to them to ensure that the primary researcher understood the meaning.

The researcher made field notes during and after the focus groups and kept a research diary for documenting personal reflections and important decisions, including the reasons for these.

### Analysis

The data collected during the focus groups were analysed using thematic analysis according to the 6-step approach of Braun and Clarke.^19^ The primary researcher was trained in coding and acted as the only coder. Each step in this process was discussed with and verified by the co-researchers. The field notes kept by the primary researcher to facilitate the understanding of the data generated were used for triangulation of the data.

### Ethical considerations

Ethical clearance was granted by the Health Research Ethics Committee of the Faculty of Medicine and Health Sciences of Stellenbosch University (HREC reference number: S18/05/110), the MBChB programme committee and the DOP. Institutional approval was also obtained. Participation was completely voluntary and informed consent was obtained from each participant. Data was stored on a password protected computer. The identities of the participants were not disclosed to anyone outside of the focus groups.

## Results

By examining the transcripts and triangulating the data with that of the field notes, three main themes were identified. These were ‘learning in the clinical context’ (with two subthemes, namely, ‘clinical environment’ and ‘patient interviews’), ‘gaining knowledge’ and ‘generational needs’.

### Theme 1: Learning in the clinical context

This theme represents participants’ perceptions of the learning activities and experiences in the various clinical placements. Although interviewing patients is part of the learning activities in the clinical environment, it constitutes a major component of clinical training in this discipline and will, therefore, be discussed separately to the rest of the students’ perceptions of the clinical environment in general.

#### Sub-theme 1: Clinical environment

Both focus groups voiced the importance of the nature of the clinical environment. They appreciated a friendly and welcoming atmosphere where they were actively included in the ward programmes and given a measure of responsibility. They enjoyed working with the doctors and the rest of the multidisciplinary team and felt valued when treated as peers.

‘The placement was nice. I thought it was a very nice ward and it is a very friendly environment and atmosphere.’ (Participant 2, Male, Focus Group 2)

Ward doctors with whom they worked were reported to be valuable role models and they strongly communicated the need for access to, and regular contact with, the placement’s doctors and consultant.

‘The doctor deals with pressure so well and he is very engaging.’ (Participant 4, Female, Focus Group 2)

‘You could just knock on her door and just tell her anything that you saw that was interesting or whatever and just ask her questions, she was very accessible.’ (Participant 7, Female, Focus Group 2)

For most of the students, working in the wards and observing the multidisciplinary team were also seen as an advantage for their learning. They could see the benefits of interacting with the team, both in learning from them and in seeing what their roles are and learning how a doctor relates to the team members in referring patients and receiving feedback.

‘I could actually see that the other members of the MDT play a pivotal role. The doctor regulates the medication, but these people actually,… they actually form for me the psycho-social part of it more, they actually do most of the hard work for the patients.’ (Participant 1, Male, Focus Group 1)

Participants reported that they learnt most when they were actively included in ward activities. Although they do not seem to want to take initiative in generating learning opportunities, if told to do something, they were more than willing to and even preferred this.

‘I actually felt quite included, they have a lot of ward rounds and they would actually ask if I saw the patient as well, what did I think or what was my comments?’ (Participant 4, Female, Focus Group 1)

Not only did they prefer to be included in activities, they also felt that being given a measure of responsibility added to their learning and to their insight into the responsibilities of being a doctor.

‘What we enjoyed the most was that you were treated as colleagues and not as students and we were given a lot of responsibility but at the same time they were always there to help us.’ (Participant 7, Female, Focus Group 2)

#### Sub-theme 2: Patient interviews

The participants found the activity of seeing and interviewing patients in the ward to be a positive experience.

‘I feel that we do gain valuable experience and get a feel for the patients.’ (Participant 6, Female, Focus Group 1)

However, there was a strong sense amongst the participants that learning took place only if the case could be discussed with a doctor. Both groups indicated that, after seeing patients, they were often unsure whether their findings were correct and that discussion with a more experienced person would be helpful to enable them to come to conclusions, such as making a diagnosis and deciding on a management plan.

‘… [*B*]ut you can see all the patients you want, but if no one is correcting you … or correcting your interpretation at all so you might be doing it incorrectly.’ (Participant 2, Male, Focus Group 2)

Despite the insight into the benefits of interviewing patients, this activity seemed to be experienced as being tedious. The participants felt that their learning was sufficient without seeing every patient or spending extended periods of time in the ward. They also felt that more time in wards did not deepen their learning, rather it became monotonous and uninteresting. The participants regarded the interviewing of patients as part of the routine ward work to be performed and not for the opportunity that it could be to gain experience. It could be concluded that the students did not make full use of the time or experience available to them.

‘I don’t need to go to see the whole ward now to get the experience, I just need to see like three different types of diseases.’ (Participant 3, Female, Focus Group 2)

Some students indicated that they would have interviewed more patients if they had been given some kind of incentive or were explicitly told to do so.

‘If we didn’t have to do the patient write-ups or patient presentations, we wouldn’t have even seen patients.’ (Participant 8, Female, Focus Group 2)

### Theme 2: Gaining knowledge

This theme relates to the participants’ opinions regarding theoretical knowledge acquisition. Although the focus of the PLCR is on clinical training, the participants expressed the need to gain more theoretical knowledge. Theoretical knowledge that was gained in previous psychiatry training phases needed to be reviewed and this revision was deemed especially necessary to help conceptualise patients interviewed in the current placements.

‘Proper formal learning must take place every single day that is structured, that is scheduled, by a consultant or by a registrar.’ (Participant 3, Female, Focus Group 1)

Most of the participants reflected that the time of studying the textbook was more valuable to them than the time spent in the clinical environment.

‘Are we going to spend more time in the ward, trying to see more patients or go home and start studying out of the text book from which all our questions is being asked from?’ (Participant 1, Male, Focus Group 1)

### Theme 3: Generational needs

This theme relates to the participants’ expectations which could be attributed to their generational needs. There is a clear need that their time should not be wasted, rather it should be respected.

‘So I think it is good if you have a set system where you can complete all your work, then you may leave and not keep us ransom till 4 o clock.’ (Participant 3, Female, Focus Group 2)

The concept of fairness was prominent during the focus groups. Many students verbalised the need to be treated fairly and wanted structures put in place to ensure this. One of the main issues regarding fairness related to the doing of ward work. They expressed feelings around being misused and did not consider this as part of the learning process.

‘Your purpose there is to learn, not to do the MO’s work.’ (Participant 5, Female, Focus Group 1)

Furthermore, a clear sense was felt about disparity between the different clinical placements.

‘You get home at 5 and you know your colleagues are getting home at 12 or 1 every day, and you didn’t get that, it’s very hard not to feel like you’ve been handed the short straw.’ (Participant 1, Male, Focus Group 1)

‘Some are getting more teaching than others and it’s not fair.’ (Participant 4, Female, Focus Group 1)

It was voiced that fairness and protection against possible misuse could be ensured if structures were in place in all settings so that they themselves, as well as the other role players, have a clear understanding of the students’ role.

‘We must have a meeting with every single role player and the role is discussed, what is the role of the student, the nurses, the doctors so we can all know what we are there for.’ (Participant 4, Female, Focus Group 1)

In addition to fairness they also wanted to be treated with consideration, especially regarding the amount of pressure that is put on them and the levels of anxiety that this provokes.

‘… [*A*]nd if it is a practice environment you are going to learn more and be more ready for the test, because if you put a mark on that experience you are going to be so frazzled. You need to make it a safe learning environment where we can make mistakes.’ (Participant 2, Male, Focus Group 1)

The participants also asked for more consideration in terms of their personal needs.

‘The doctors don’t feel any sympathy or empathy towards you.’ (Participant 3, Female, Focus Group 1)

‘You can’t just take the day off.’ (Participant 3, Female, Focus Group 2)

## Discussion

This study explored student perceptions of an undergraduate psychiatry rotation, and the findings generated will be used to inform the curriculum renewal process that is currently being undertaken by the DOP at FMHS. The goal of this curriculum renewal is to ensure the graduation of more rounded primary care physicians. In general, the opinion from the participants of the focus groups was that the PLCR provided them with a good learning experience in psychiatry. They were particularly referring to the clinical environment and working with the multidisciplinary team and felt that this contributed to a positive experience. However, some important points were raised about the current curriculum, and several suggestions were made to potentially improve the current practice. Keeping in mind that these perceptions are from a group of millennials, some interesting insights arise especially when considering how this generation has been influenced by the world around them and the way in which they were raised.

Being millennials, our sample of students are expected to have had a less authoritative upbringing than most of their teachers and were active participants in their own households since an early age.^20^ They grew up to question the world around them. These students now have a need to know what they are doing and why they are doing it.^20^ They want an explanation for why things are performed a certain way, as also expressed by our focus groups, and do not just want to accept the way things are performed.

As per Kolb’s experiential learning theory,^5^ the students were placed in the clinical environment to have a ‘concrete experience’. Many participants commented that this was a positive experience as the atmosphere was friendly and welcoming. They felt that they learnt a lot from the multidisciplinary team and enjoyed partaking in the routine ward activities. However, the students did not seem to realise that being closely involved with the clinical environment helps them to develop competencies such as being professionals, communicators, collaborators, leaders and managers, health advocates and scholars.^21^ Also, they appeared unaware that the doctors as role models^22^ could help them to learn how to better deal with conflict, responsibility and pressures in the workplace. These findings show that if we want students to buy into learning through ‘concrete experience’, then it is prudent that we explain the rationale behind the need for them to engage both in the clinical environment and with patients. We as teachers need to explicitly identify the ‘concrete experience’^5^ as such to students.

The participants of the focus groups were of the opinion that they do benefit from interviewing patients, but they found it challenging to link the clinical presentation of the patient to the theory from the textbook. Transferring knowledge to practice is a skill that develops through reflection and is a necessary step towards developing critical thinking.^23^ According to Kolb’s learning cycle,^5^ reflection is an essential part of the learning process. From the focus groups, it appears that this is not a natural process for students and they strongly voiced the need for regular contact with doctors to give them a measure of direction in doing so.

As most of our students form part of the millennial generation, it is possible that many grew up in a very child-focussed world exposed to the so-called ‘helicopter parents’.^24^ These parents are overprotective of their children and overinvolved in their lives. Students who were exposed to such an upbringing may now, upon coming into the clinical environment, potentially have the need for the same ‘anxious hovering’^24^ from their teachers. We as teachers can meet this need by ensuring accessibility and approachability or even take it a step further by providing a mentoring programme. The ideal should be a space where students are allowed to grapple with problems but could be guided towards solutions by their teachers.^24^ Participants expressed very specific learning needs, which included role clarification and clear guidelines on expectations for clinical placements.

In focus groups, routine ward activities and follow-up patient interviews were reported to be perceived as the work of the permanent clinical staff; and students who commented on this felt that it was unfair to be expected to participate in these activities. It is possible that teachers from the previous generation may interpret this to reflect apathetic, disinterested, tuned out and selfish attitudes, but this might well be an erroneous perception. Chelsea Clinton^25^ previously commented that this particular assumption about millennials is unfounded.

Many millennials are quick to emphasise that a ‘work–life balance’ is important to them and that they do not see work as ending at 16:00 and personal life starting at this time. Rather, they see the two on a continuum.^26^ Where this is the case, it could translate into a need to not wanting their time to be wasted and to be afforded the same level of respect, despite being younger or more junior.

They also want consideration for their personal needs^20^ and appreciate a learning atmosphere that is relaxed, with minimal anxiety and pressures and with the flexibility to self-determine the pace of learning. Clinical environments, because of the nature of the work that needs to be performed, often do not afford students the freedom to take charge of their own learning. It would, thus, be good to critically reflect on ways of creating more awareness in this regard but also collaboratively seek more opportunities for students to do so.

Curriculum design is influenced by the interplay between many factors, not least of which would be the values, beliefs and choices of the teachers who take the lead in the process.^3^ When evaluating and renewing a curriculum, it is imperative to acknowledge the influence of generational differences between teachers and their students, otherwise it is unlikely to be appropriately factored in when critical decisions are made. Whilst it will not be possible to implement all suggestions, the students shared many valuable insights. Our findings clearly support the notion that learner input and the critical evaluation thereof play a vital part in the process of developing a curriculum that will come close to meeting the standards expected by teachers as well as achieving acceptance from learners.^20^

Our study is potentially limited by the fact that it was a small-scale, single-site study that only explored the perceptions of students who were self-selected on a first-come-first-serve basis. Also, the primary researcher, who conducted the focus group discussions and subsequent analysis, is a teacher of the participants, and we acknowledge the bias connected with an insider researcher. However, interesting insights were gained in terms of aligning the students’ perceptions within the context of the generational gap, a concept that can be universally applied and, thus, can be regarded as a strength.

## Conclusion

During the rotation evaluation process, our study participants offered many suggestions for change but still endorsed that the PLCR does provide them with a good learning experience in psychiatry. Whilst it will not be possible to implement all suggestions during the curriculum renewal process, the students’ perceptions led to many valuable insights, particularly when framed within the context of the millennial generation’s needs. Taking the generational gap into consideration, future research with a specific focus on clinical teachers’ perceptions is needed. Understanding both the needs of teachers and students, whilst not compromising on quality, would likely be the best way to bridge the generational gap.
